# Monoamine oxidase inhibitors *l*-deprenyl and clorgyline protect nonmalignant human cells from ionising radiation and chemotherapy toxicity

**DOI:** 10.1038/sj.bjc.6601361

**Published:** 2003-11-11

**Authors:** C B Seymour, C Mothersill, R Mooney, M Moriarty, K F Tipton

**Affiliations:** 1 Radiation and Environmental Science Centre, Dublin Institute of Technology, Kevin St, Dublin 8, Ireland; 2 St Luke's Institute for Cancer Research, Rathgar, Dublin 6, Ireland; 3 Department of Biochemistry, Trinity College, Dublin 2, Ireland

**Keywords:** radiation protection, monoamine oxidase inhibitors (MAOIs), *l*-deprenyl, clorgyline, apoptosis

## Abstract

*l*-Deprenyl (*R*-(−)-deprenyl, selegiline) is an inhibitor of monoamine oxidase-B (MAO-B) that is known to protect nerve cells from a variety of chemical and physical insults. As apoptosis is a common mechanism of radiation-induced cell death, the effect of *l-*deprenyl on the survival of cultured cells and tissue explants was studied following exposure to gamma radiation. The results obtained were compared with the effects of the less-selective MAO-B inhibitor pargyline and the MAO-A inhibitor clorgyline. *l*-Deprenyl at a concentration of 10^−9^ M protected the nontumorigenic cell line (HaCaT) and normal human urothelial explants from the effects of cobalt-60 gamma radiation, but did not protect tumorigenic human cell lines HaCaT-ras, HPV-transfected human keratinocytes (HPV-G cells), or PC3. Human bladder carcinoma explants were not protected. Clorgyline showed a smaller protective effect of normal cells, whereas pargyline had no effect. Radiation-induced delayed effects (genomic instability measured as delayed cell death) were prevented in normal cells by *l*-deprenyl but, interestingly, deprenyl appeared to increase the amount of delayed death in the tumorigenic cell lines. Studies using *l*-deprenyl prior to the exposure of nonmalignant cells to cisplatin showed that cell death due to this agent was also reduced. Treatment of cultures of nontumorigenic cells with *l-*deprenyl or clorgyline significantly increased the levels of the protein Bcl-2 following irradiation, but there was no such effect on the already-elevated levels of this protein in the tumour samples. Since the Bcl-2 has been shown to be an inhibitor of apoptosis or programmed cell death, this would imply that the protective effects of *l*-deprenyl and clorgyline involve activation of antiapoptotic pathways within the normal cell. This hypothesis is supported by data showing reduced levels of apoptosis in HaCAT cells and in normal bladder explant cultures following treatment with *l*-deprenyl.

Protection of normal tissues from damage during radiotherapy is a key goal of experimental radiotherapy and a key problem in patient treatment. Most chemical approaches aim to sensitise the tumour, but it is equally valid to seek radioprotective drugs which will selectively protect the normal cells. As an approach to this, our group looked at agents already in clinical use, which aim to reduce or prevent death or damage of cells during degenerative disease processes. In this paper, we report results using monoaimine oxidase inhibitors. Since the biochemistry of these and their function in protecting cells against death is unlikely to be familiar to oncology specialists, the background is reviewed somewhat extensively in this introduction.

Monoamine oxidase (EC 1.4.3.4; MAO) enzymes catalyse the oxidative deamination of a range of monoamines, including the catecholamines and serotonin. The enzyme exists in two forms, MAO-A and MAO-B (for reviews, see Yu, 1986; O'Brien and Tipton, 1994), which are encoded by distinct gene loci on the X-chromosome (Shih, 1991).

*l*-Deprenyl (*R*-(−)-deprenyl, selegiline, eldepryl) is an irreversible inhibitor of MAO-B, which has been used as an adjunct to reduce the oxidation of dopamine, in the L-DOPA-treatment of Parkinson's disease (see Birkmayer *et al*, 1975). L-deprenyl has been shown to protect against neuronal damage resulting from other chemical and physical insults, and it was suggested that the actions of this compound might be better described as neuronal rescue rather than neuronal protection (Salo and Tatton, 1991; Tatton and Greenwood, 1991). The mechanisms involved of this neurorescue effect are unclear. *l*-Deprenyl, and some other MAO-B inhibitors, bind to the apoptosis-regulating protein glyceraldehyde-3-phosphate dehydrogenase (Kragten *et al*, 1998), and also stimulate the expression of a number of antiapoptotic gene products (see Tatton *et al*, 2002), as well as the amine synthesis enzyme L-aromatic amino-acid decarboxylase (Li *et al*, 1992). *l*-Deprenyl at concentrations too low to inhibit MAO-B (<10^−9^ M) prevents PC12 cells from undergoing apoptosis induced by trophic factor withdrawal in a process that requires new protein synthesis (Tatton *et al*, 1994a,1994b). This has led to the suggestion that *l*-deprenyl conveys its protective effects via the activation of antiapoptosis pathways within the cell, and that this neuroprotection is independent of MAO-B inhibition.

*l*-Deprenyl may be involved in regulation of the cell cycle, where it could possibly prevent the senescence of astroglial cells, which presumably act by providing trophic factor support to the nerve cells (Shimazu *et al*, 1993). Among the other neuroprotective mechanisms that have been proposed are an antioxidant effect (Chiueh *et al*, 1994) and the preservation of mitochondrial membrane potential, and an associated inhibition of mitochondrial permeability-transition pore opening (see Wadia *et al*, 1998; Zhang *et al*, 1999). Although studies in relation to the protective effects of *l*-deprenyl have concentrated on nerve cells or related systems, such as dopaminergic PC12 cells (Tatton *et al*, 1994a,1994b), there appeared to be no *a priori* reason to believe that its protective or rescue effects might not be more general. In order to investigate such a possibility, we have investigated the effects of *l*-deprenyl, pargyline and clorgyline on the survival of cultured cells and tissues following exposure to *γ*-radiation from a radiotherapeutic cobalt-60 (Co-60) source.

## EXPERIMENTAL METHODS

### Cell lines and culture conditions

Four cell lines were used in this study. Wild-type HaCaT are considered to be immortal but nonmalignant; HPV-G, HaCaT-ras and PC3 cells are generally considered to be tumorigenic. HaCaT-ras and PC3 form malignant tumours in immune-suppressed mice and HPV-G cells form benign nodules. The HaCaT cell line is a normal human keratinocyte cell line derived from human skin keratinocytes spontaneously transformed *in vitro* during long-term incubation of a primary culture under selected culture conditions (Boukamp *et al*, 1999). It was obtained as a kind gift from Dr P Boukamp (DKFZ, Heidelberg University, Germany), who also supplied us with the H-ras-transfected variant of HaCaT cells. The HPV-G cell line is a human immortal keratinocyte cell line that was derived from human neonatal foreskin transfected with the HPV-16 virus in order to cause immortalisation (Pirisi *et al*, 1987). It was obtained as a kind gift from Dr J Dipaolo (NIH, Bethesda, MD, USA). The PC3 cell line is a human prostate adenocarcinoma cell line established from a grade 4 prostatic adenocarcinoma from a 62-year-old male Caucasian, and was obtained from the European collection of cell cultures (ECACC). All the cell lines were maintained at 37°C in Dulbecco's MEM: Ham's nutrient mixture (F12) 1 : 1 (Gibco Biocult, UK) supplemented with 10% foetal calf serum, 50 IU ml^−1^ penicillin, 50 *μ*g ml^−1^ streptomycin solution, 2 mM L-glutamine, 1 *μ*g hydrocortisone and 12.5 ml HEPES buffer (1 M), all from Gibco Biocult, UK.

### Assay of cell survival and frequency of lethal mutations (delayed cell death)

The standard clonogenic assay (Puck and Marcus, 1956) with the lethal mutation extension (Seymour *et al*, 1986) was used for all cell lines. A healthy confluent flask of cells was trypsinised using 0.25% w v^−1^ trypsin solution and 1 mM versene solution in a 1 : 1 mixture. The resulting cell suspension was counted using a Coulter counter (Corning model Dn). Appropriate cell numbers were then plated in 5 ml of culture medium. At the appropriate times post irradiation when the irradiated cells produced colonies containing approximately 200 cells, half of the cultures were stained with carbol fuchsin (Ziehl Nielson 1 : 15). The number of macroscopic colonies was counted and the initial radiation survival determined. The rest of the cultures were grown to confluence, subcultured and replated without further irradiation to measure delayed cell death (lethal mutations) in the progeny.

### Explant culture technique

Biopsies of normal urothelium were obtained from patients undergoing reconstructive or investigative surgery for benign conditions. These were set up as explants, using the techniques described by Mothersill *et al* (1988). Bladder tumour biopsies were obtained during clinical investigations for suspected malignancy. The malignant nature of these biopsies was subsequently confirmed by the histopathology. All were confirmed as low-grade transitional cell carcinomas (TCC). The tissue was placed in sterile physiological saline immediately upon removal from the patient, and placed in a refrigerator until it could be processed. Cultures were normally established within 6 h, although growth was achieved successfully even after 24 h storage at 4°C. The biopsies were divided into approximately four explant-sized pieces, and were then transferred into 0.25% w v^−1^ trypsin solution (Gibco Biocult, UK) containing 10 mg ml^−1^ collagenase type IV (Sigma, USA) and incubated for 30 min at 37°C. After this, the partially digested pieces were plated individually as explants in 25 cm^2^ 40-ml volume tissue culture flasks containing 2 ml RPMI 1640 medium (Gibco Biocult, UK), supplemented with 7% foetal calf serum, 3% horse serum, 5 ml 100 × penicillin/streptomycin solution (all from Gibco Biocult, UK), 1 *μ*g ml^−1^ hydrocortisone (Sigma), 0.1 ng ml^−1^ epidermal growth factor (Sigma) and 100 IU ml^−1^ insulin (Actrapid Novo Nordisk, Denmark). When the explants had attached, usually within 2–3 days, the cultures were transferred into Clonetics Keratinocyte Growth medium (Clonetics Corp., San Diego, CA, USA). This medium is a serum-free formulation containing insulin, hydrocortisone, epidermal growth factor, bovine pituitary extract and the antibiotics, ampicillin and gentamycin. Cultures were maintained in this medium with no medium changes for 2–3 weeks (Mothersill *et al*, 1995). The tumour biopsy material was processed using the same method.

### Monoamine oxidase inhibitors (MAOIs)

*l*-Deprenyl was obtained from Semat Technical Ltd (St Albans, Herts, UK) and clorgyline (*N*-methyl-*N*-propargyl-3-(2,4-dichlorophenoxy)-propylamine hydrochloride) and pargyline (*N*-methyl-*N*-2-propynylbenzylamine hydrochloride) were both supplied by Sigma. All MAOIs were made up to the desired concentrations in deionised water and were administered to each cell or explant culture 6 h prior to irradiation. The concentrations of MAOIs used ranged from 10^−11^ to 10^−3^ M. The concentration used for neurorescue in the clinic is 10^−9^ M. The volume of MAOI added to each cell or explant culture was always 100 *μ*l. In the control cultures, where no MAOI was added, 100 *μ*l of deionised water was added instead of the MAOI, in order to keep the volume of cell culture medium constant throughout the experiment.

### Irradiation

Cultures of cell lines were treated 8 h after plating. Explants were irradiated 3 days after plating and after transfer into serum-free medium. Irradiation was performed at room temperature using a Co-60 teletherapy unit delivering approximately 1.8 Gy min^−1^ at 80 cm source-to-flask distance. The doses chosen were 0.5 and 5 Gy. All treated flasks and sham-irradiated controls were returned immediately to the CO_2_ incubator and left undisturbed for 9 days to allow colony formation. Cell lines were generally stained with carbol fuchsin for assessment of clonogenic survival but, in some cases, colonies were fixed in 10% unbuffered formalin to allow immunocytochemical investigations to be done. Explant cultures were maintained for 2 weeks and then fixed with formalin and studied using immunocytochemistry.

### Immunocytochemistry

At appropriate times (9 days for the cell lines or 2 weeks for the explants), cultures were fixed in formalin for 24 h, then stored in PBS at 4°C for not more than 1 week before staining. The plastic flasks were then cracked open using pliers and the explant culture, or cell line clones were processed on the flask base as for specimens mounted on glass slides. This method preserved the spatial organisation and distribution of the staining in the culture. Immunostaining was performed using Vectastain ABC® kits (Vectastain Corp, Burlingame, CA, USA) with diaminobenzidine (DAB) as the chromogen. The primary antibody used to detect Bcl-2 expression was the anti-Bcl-2 antibody (DAKO Laboratories, Bucks, UK). This antibody was divided into aliquots on receipt and stored at −20°C until required. Positive and negative controls were run with each batch of cultures. The positive control for Bcl-2 expression consisted of a Bcl-2-positive lymphoma section. Negative controls routinely included with each run were the same cell lines or explants run with no primary antibody. In all cases, cultures were counterstained using Mayer's haematoxylin.

### Quantification of Bcl-2 protein expression

The expression of Bcl-2 was quantified in the cultures using image analysis (Leica system). The fields for analysis were chosen along two transects drawn at right angles to each other across the explant culture. This method ensured that any differences between cells at the centre and edge of the explant were accounted for, and that any evidence of clonal expression originating from specific irradiated cells would be identified. A cell was designated as positive if the staining was equal to or greater than the positive control. The cell number was determined by measuring the area of the explant outgrowth and the number of cells in 10 replicate 1 mm^2^ sample areas chosen at random from the culture. For cell lines, positive cells were counted in 10 randomly chosen clones at 7–9 days postirradiation or in 100 randomly chosen microcolonies, where 48 h data were being collected. In each case, counts were made in each of three replicate cultures and expressed as a function of the total cells counted in the clone or microcolony.

### Measurement of apoptosis in explant cultures and in HaCaT microcolonies

Apoptosis was scored in the explant outgrowth cultures by counting the numbers of apoptotic cells in 10 randomly chosen 100 *μ*^2^ fields at 30 min before, and at 48 h and 14 days postirradiation. Identification of apoptotic cells was performed using the criteria defined in Lyng *et al* (2002) and the results were confirmed using electron microscopy.

In the clonogenic cell lines (HaCaT and HaCAT-ras), microcolonies were examined at 48 h postexposure to irradiation for signs of apoptosis. In all, 100 microcolonies per dish were scored and the total apoptotic cells over total cells counted was expressed as a percentage. Previous data from our group have validated these methods (Mothersill *et al*, 1995).

### Statistical analysis

All experiments were repeated three independent times. Within each experiment, assays were set up in triplicate. The results are the mean±s.e.m. for the three independent experiments. Significance was assessed using the Student's *t*-test.

## RESULTS

### Effects of MAOIs on the relative cell survival of normal uroepithelial and bladder tumour explants exposed to Co-60 *γ* radiation

Figure 1

**Figure 1:**
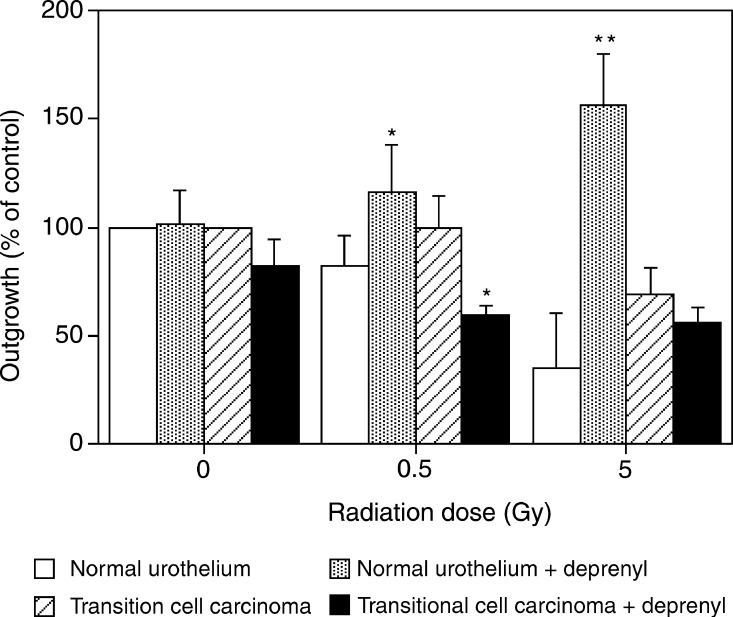
Effects of pretreatment with 1 nM *l*-deprenyl on the radiation response of human normal and malignant bladder explants. The area of the outgrowth was measured on day 14 postirradiation and the treated outgrowth area is expressed as a percentage of the control (mean±s.e.m. for five patients per group). Absolute values for the normal control were 460±120 mm^2^ and for the tumour 246±60 mm^2^. ^*^P<0.05, ^**^P<0.01.

shows the effect of deprenyl, at a concentration of 10^−9^ M, on the survival of normal human urothelial explants exposed to 0, 0.5 or 5 Gy of Co-60 gamma radiation; *l*-deprenyl caused a significant increase in survival after 0.5 and 5 Gy.

The effect of *l*-deprenyl on human bladder tumour explants exposed to 0, 0.5 and 5 Gy of Co-60 *γ* radiation is also shown in Figure 1. At all radiation doses examined, *l*-deprenyl at a concentration of 10^−9^ M, which was radioprotective for normal tissue explants, showed no radioprotective effect for tumour explants. In contrast, there is a trend for tumour explants to be sensitised by exposure to *l*-deprenyl, although this is only statistically significant at the 0.5 Gy dose.

### Effects of l-deprenyl and clorgyline on the initial radiation survival of four cell lines, exposed to Co-60 *γ* radiation

Table 1

**Table 1:** Effects of *l*-deprenyl (D) and clorgyline (C) on the survival of four clonogenic cell lines

		**Plating efficiency (% of control)**			
**Radiation dose (Gy)**	**MAOI**	**HaCaT cells**	**HaCaT-ras cells**	**HPV-G cells**	**PC3 cells**
0	0	100 (28.6±2.5)	100 (56.7±4.4)	100 (23.4±2.1)	100 (11.9±1.3)
0.5	0	81.3±5.4	100±5.2	65.7±3.3	80.4±3.4
5	0	17.4±1.1	20.6±3.3	19.9±1.7	17.5±1.2
0	D	154±3.8	112±3.1	120±3.8	102±5.6
0.5	D	165±3.5	98±5.2	103±4.8	89.2±7.7
5	D	82±4.9	17.9±3.7	21.0±2.1	20.4±3.6
0	C	170±5.7	90.1±4.0	97.5±2.7	96.6±2.7
0.5	C	149±6.6	77.3±3.5	99.2±3.7	75.1±4.8
5	C	79.0±4.3	12.5±3.4	20.0±1.7	19.0±2.3

All cell lines were set up in a medium containing the indicated monoamine oxidase inhibitor (MAOI) at a concentration of 1 nM and irradiated 6 h later. All plating efficiencies are expressed relative to the control, which received no radiation, D or C.

shows the initial radiation survival of the four cell lines, HaCaT, HaCaT-ras, HPV-G and PC3 cell lines, exposed to 0, 0.5 or 5 Gy of Co-60 *γ* radiation with *l*-deprenyl or clorgyline at concentrations of 10^−9^ M, which is the concentration used in the clinic. The results show that *l*-deprenyl acted as an effective radioprotector for HaCaT cells irradiated to 0.5 or 5 Gy. In some experiments, higher concentrations of *l*-deprenyl were tested. The radioprotective effect was significantly (*P*<0.05) greater after 10^−9^ M than after 10^−5^ M *l*-deprenyl (data not shown). Clorgyline at a concentration of 10^−9^ M also acted as an effective radioprotective compound at these radiation doses. Examination of the control PE data in the presence of these agents shows that the plating efficiency of the cells is higher, suggesting that death of cells is reduced even in the absence of radiation. It is also apparent on analysis of the data that the radioprotective effect is increased with increasing dose. That is, the greater the radiation dose, the greater the radioprotective effect.

With HPV-G cells, which have compromised but wild-type p53 due to the presence of the E6 protein of the HPV virus, *l*-deprenyl or clorgyline at a concentration of 10^−9^ M produced no radioprotective effect at either 0.5 or 5 Gy doses. The data for the PC3 human prostate carcinoma cell line also demonstrate the absence of *l*-deprenyl-induced radioprotection in a tumour cell line.

Figure 2

**Figure 2:**
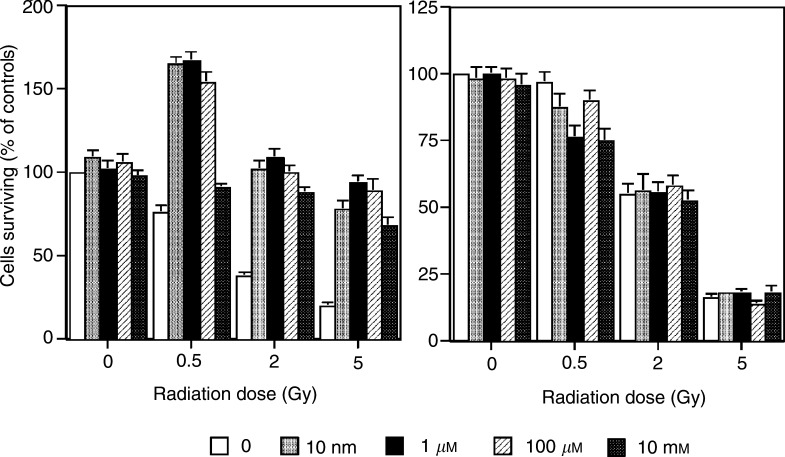
Effects of varying concentrations of *l*-deprenyl and varying radiation doses on the % of HaCaT or HaCaT-ras cell colonies surviving treatment. Cells were plated in medium containing 1 nM *l*-deprenyl and irradiated 6 h later. Absolute control plating efficiency values were 34.5±2.7 for HaCaT cells and 56.7±3.7 for HaCaT-ras cells.

shows clonogenic survival data for HaCaT cells and the H-ras-transfected variant exposed to a range of *l*-deprenyl concentrations (10^−5^–10^−11^ M) prior to a range of radiation doses (0.5–5 Gy). There were no protective effects of *l*-deprenyl using the ras-transformed line, whereas protection was observed with the normal line. The *l*-deprenyl dose–response for the normal keratinocytes was stable at doses lower or equal to 10^−5^ M. A high 1 mM dose was also tested, but was toxic, and, as no colonies formed, the data were excluded from the figure.

### Effects of *l*-deprenyl and clorgyline on induction of lethal mutations (delayed reproductive death)

Table 2

**Table 2:** Delayed reproductive death (clonogenic survival of subcultured cells) in distant progeny of cells whose progenitors were exposed to 1 nM*l*-deprenyl or 1 nM clorgyline

		**Survival of progeny (% of control)**		
**Radiation dose (Gy)**	**MAOI**	**HaCaT cells**	**HaCaT-ras cells**	**HPV-G cells**
Control 0	None	100 (27.0±3.1)	100 (96.1±3.3)	100 (16.0±1.1)
Control 0.5	None	100 (22.2±2.7)	100 (57.2±3.9)	100 (11±0.6)
Control 5.0	None	100 (6.5±0.4)	100 (57.9±2.9)	100 (10±0.4)
0	*l*-Deprenyl	138±7.3	60.0±4.1	120±8.9
0.5	*l*-Deprenyl	127±6.4	120±10.3	196±0.7
5.0	*l*-Deprenyl	200±13.1	90.1±3.7	126.3±6.7
0	Clorgyline	104±5.4	64.3±2.8	75.6±3.4
0.5	Clorgyline	123±3.8	110±7.1	116±8.2
5.0	Clorgyline	133±6.1	90.6±2.8	126±7.7

The clonogenic assay was performed on cultures that had undergone at least 15 population doublings postexposure.

shows the effect of *l*-deprenyl on induction of delayed cell death/lethal mutations in progeny of cells exposed to irradiation. HaCaT, HaCaT-ras and HPV-G were exposed to 0, 0.5 or 5 Gy of Co-60 gamma radiation with *l*-deprenyl or clorgyline at a concentration of 10^−9^ M. The exposed cells were grown to confluence and then subcultured without further treatment with radiation, and without further addition of *l*-deprenyl to the cells. The survival of the progeny was examined after at least 15 population doublings. The values for plating efficiency below 100% show that delayed cell death is occurring. The values above 100% are associated with protection from delayed death or proliferation-stimulating delayed effects. *l*-Deprenyl or clorgyline treatment of HaCaT progenitor cells prior to irradiation protected progeny cells originally irradiated at 0.5 Gy from delayed death. When the progenitor cultures were treated with 10^−9^ M *l*-deprenyl and then irradiated to 5 Gy, the progeny of the irradiated cells shows a large antidelayed death radioprotective effect. Clorgyline also protected progeny cells; however, this protective effect was less than that found with *l*-deprenyl. Data for the tumorigenic cell lines show much less protection either with *l*-deprenyl or clorgyline in cultures irradiated to 0.5 or 5 Gy. In fact, the survival of *l*-deprenyl- or clorgyline-treated tumour cell line progeny was significantly decreased by the treatment alone (*P*<0.01).

### Effects of *l*-deprenyl on survival of HaCaT and HaCaT-ras cells treated with cisplatin

**Figure 3:**
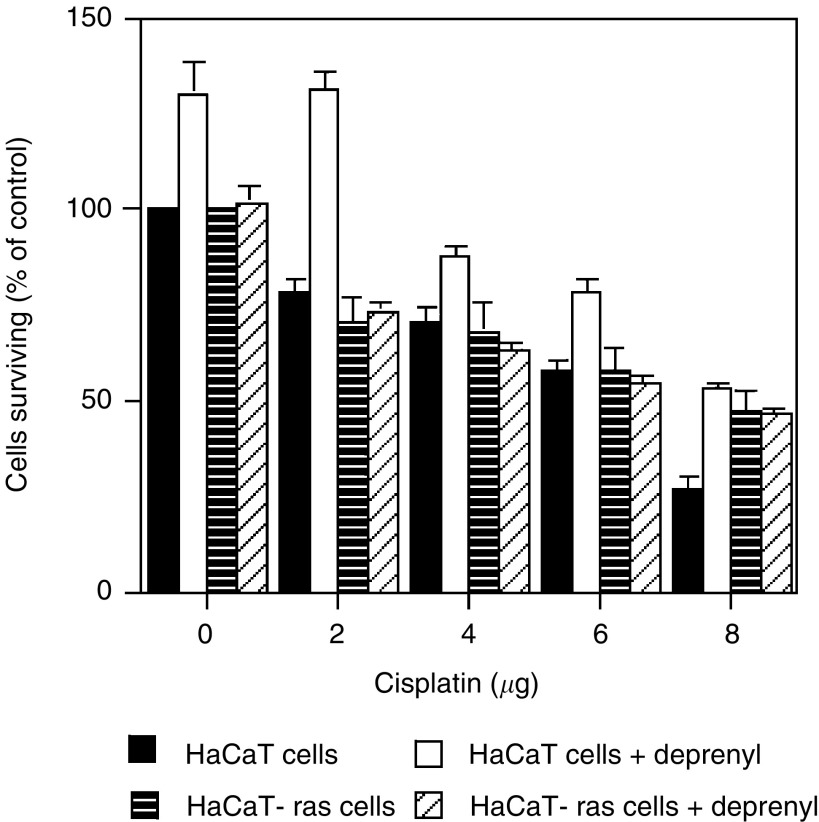
Clonogenic survival for HaCaT and HaCaT-ras cells treated with cisplatin (*μ*g ml^−1^) for 1 h. Cells were plated in medium containing 1 nM *l*-deprenyl and exposed to cisplatin 16 h later. Absolute control plating efficiencies were 23.6±1.3 for HaCaT cells and 52.2±2.1 for HaCaT-ras cells.

Most cancer therapy involves chemotherapy as well as, or instead of, radiotherapy; therefore experiments were done to determine whether *l*-deprenyl could protect normal cells from cisplatin toxicity. The data for the normal HaCaT cells are shown in Figure 3

. Clearly, *l*-deprenyl can protect normal cells from cisplatin toxicity. HaCaT-ras cells showed no protection from toxicity attributable to *l*-deprenyl. This effect again appears to be mainly due to the enhanced clonogenic survival of the control cells following deprenyl treatment, rather than to the active prevention of cisplatin toxicity.

### Expression of Bcl-2 protein and apoptosis

Figure 4A

**Figure 4:**
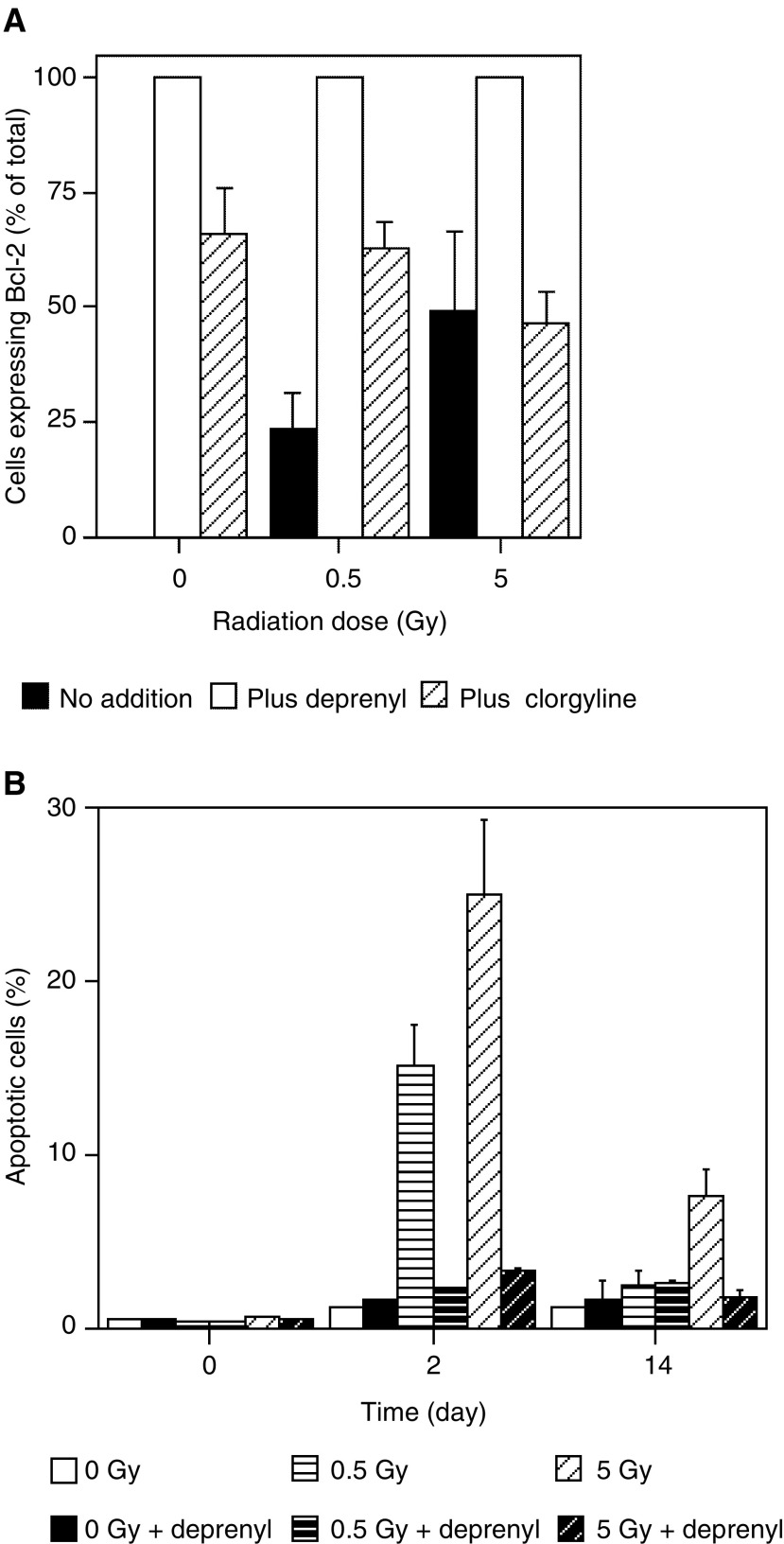
(**A**) Expression of Bcl-2 protein in normal human uroepithelial explants, treated as indicated with 1 nM *l*-deprenyl or clorgyline. The values for the tumour samples were all 100%, and are not shown. (**B**) Apoptosis postirradiation in primary urothelial cultures treated with *l*-deprenyl. Data are mean percent counts of apoptotic cells in 10 randomly selected fields per culture.

shows Bcl-2 protein expression in primary normal and tumour explant cultures at the end of the experiment (generally 14 days) postirradiation, detected using immunocytochemistry. This technique was used in preference to Western blotting, because of the very small numbers of cells which grow from primary explants and because immunocytochemistry allows spatial information to be retained and permitted us to quantify the numbers of cells expressing the protein. Owing to the small numbers of explants available from human tissue biopsies, it was not possible to study Bcl-2 expression during the growth period; however, the apoptosis data described in the next section were obtained by noninvasive methods. Treatment with *l*-deprenyl or clorgyline resulted in increased Bcl-2 expression in the normal human uroepithelial explants in all circumstances. In the bladder carcinoma explants, Bcl-2 expression was found to be 100% positive both in the presence and absence of *l*-deprenyl or clorgyline. Figure 4B shows the levels of apoptotic cells in explant cultures before being treated with 0.5 or 5 Gy Co-60 *γ*-rays, at 48 h afterwards and at the end of the experiment. The results show that preirradiation, there are only very low levels of apoptotic cells in the explant cultures. After 48 h, the level in the irradiated cells rises significantly in the radiation-only group, but remains significantly lower in the deprenyl-treated group. By the time the cultures are fixed at the end of 14 days in the explant system, the detectable levels of apoptosis have fallen but are still higher than the preirradiation level. Some experiments were done to assess effects on BAX and p53 protein expression.

Table 3

**Table 3:** Effects of monoamine oxidase inhibitors and irradiation on Bcl-2 protein expression in initially irradiated cultures of HaCaT cells treated as indicated

**Radiation dose (Gy)**	**MAOI**	**% Positive HaCaT cells**
0	None	0
0	1 nM *l*-Deprenyl	100
0	10 *μ*M *l*-Deprenyl	49±12
0	1 nM Clorgyline	56±8
0.5	None	12±4
0.5	1 nM *l*-Deprenyl	100
0.5	10 *μ*M *l*-Deprenyl	79±15
0.5	1 nM Clorgyline	69±11
5.0	None	58±13
5.0	1 nM *l*-Deprenyl	100
5.0	10 *μ*M *l*-Deprenyl	100
5.0	1 nM Clorgyline	100

shows data for the HaCaT cell line. Clearly, treatment with *l*-deprenyl leads to increased post irradiation expression of Bcl-2 in the normal cell line. In the HaCaT-ras cells, Bcl-2 was expressed in all cells in all microcolonies at 48 h and at 9 days postirradiation. *l*-Deprenyl treatment had no further effect (data not shown). In the other cell lines, expression of Bcl-2, measured at 7–9 days after irradiation, was also already elevated in all cells in all the cultures and neither *l*-deprenyl nor clorgyline treatment had any further effect (data not shown).

In order to test the hypothesis that Bcl-2 expression was directly linked to the suppression of apoptosis, experiments were done with HaCaT cells where cultures were fixed at 48 h and 7–9 days postirradiation. Apoptosis and Bcl-2 expression were then measured in the same microcolony or colony.

Immunocytochemistry was the method of choice in the cell line studies because, in order to perform clonogenic assays, only small numbers of cells can be inserted into a flask (100–500). Also, by retaining spatial information concerning the multiplicity of the microcolony, the data can be used to correlate apoptotic scores with Bcl-2 scores directly and in relation to microcolony size (a good indicator of likely survival). It is now recognised by our group that in order to correlate data concerning apoptosis and Bcl-2 expression, similar cell number : medium volumes must be used. If they are not, the results are influenced by neighbour effects involving signalling and energy substrate concentrations. Therefore, extreme care was taken to measure apoptosis and Bcl-2 in similarly seeded cultures. Measurements were done at 48 h when apoptosis was measured and at 7–9 days when reproductive survival was determined.

**Figure 5:**
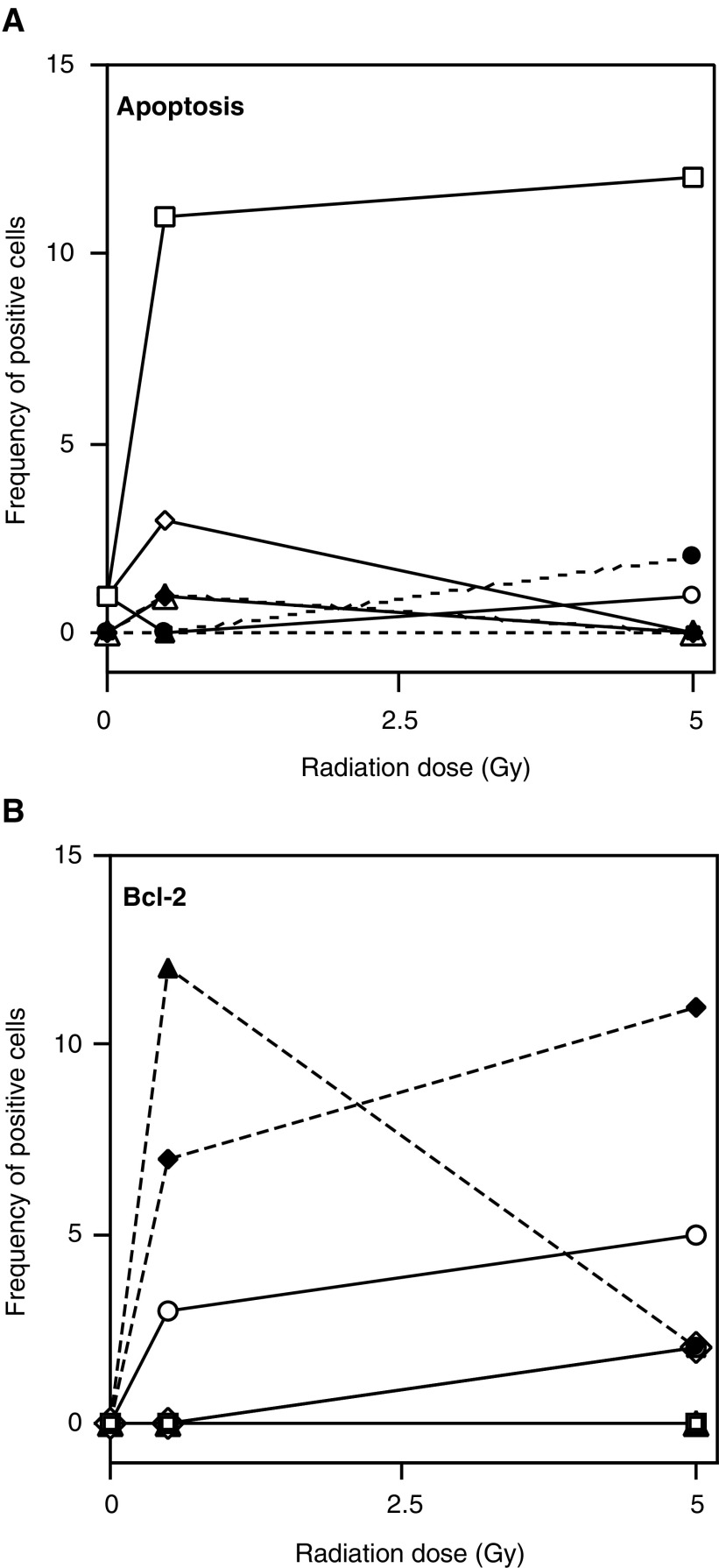
Expression of apoptosis (**A**) and bcl-2 (**B**) in HaCaT cells with and without *l*-deprenyl treatment prior to irradiation. Cultures were all seeded with 400 cells and were fixed at 48 h postirradiation to 0, 0.5 or 5 Gy Co-60 gamma rays. Apoptosis and Bcl-2 expression were scored in the same microcolonies. In all, 20 microcolonies were scored for each culture. Five replicate cultures were counted. Data were pooled due to low frequency of the end points. The figures show the relative frequency of apoptotic and Bcl-2-positive cells in the microcolonies. Open symbols, solid lines: − deprenyl; Closed symbols, broken lines: + deprenyl. Cells per microcolony: 1 (□, ▪); 2 (○, •); 3 (◊, ⧫); 4 (▵, ▴).

The results that are shown in Figure 5A and B

and Table 4

**Table 4:** Apoptosis postirradiation in colonies or potential colonies of HaCaT cells treated with *l*-deprenyl (mean percent apoptotic cells averaged over 100 colonies or potential colonies)

	**Preirradiation**		**48 h Postirradiation**		**9 Days postirradiation**	
**Treatment**	**Cells per colony**	**Apoptosis%**	**Cells per colony**	**Apoptosis%**	**Cells per colony**	**Apoptosis%**
0 Gy control	1	0	3.6±0.12	0.2±0.03	268±32	0.24±0.03
0.5 Gy	1	0	1.1±0.14	25.6±4.6	165±16	2.15±0.34
5 Gy	1	0	1.4±0.19	32.1±2.9	126±13	2.93±0.8
0 Gy+10^−9^ M *l*-deprenyl	1		3.7±0.08	0.1±0.008	374±30	1.1±0.07
0.5 Gy+10^−9^ M *l*-deprenyl	1	0	3.2±0.41	0.24±0.04	321±35	0.86±0.05
5 Gy+10^−9^ M *l*-deprenyl	1	0	2.6±1.1	1.22±0.3	274±19	0.75±0.06

confirm that Bcl-2 levels were elevated after 48 h (Figure 5) and also after 7–9 days (Table 4), when the normal cells were exposed to *l*-deprenyl. Clorgyline produced a similar effect (data not shown). In Figure 5A and B, data are also presented to show the frequency of apoptosis and Bcl-2 expression in the same microcolonies 48 h after exposure to 0.5 or 5 Gy with (Figure 5B) or without (Figure 5A) l-deprenyl. Microcolonies are grouped according to the number of cells in the microcolony. Clearly, apoptosis and smaller microcolony size dominate in the untreated group and Bcl-2 expression together with larger microcolony size dominate in the treated group. Data for the HaCaT-ras cell line were also obtained and there was no apoptosis in any of the cultures with or without treatment.

## DISCUSSION

Both *l*-deprenyl and, to a somewhat lesser extent, clorgyline at a concentration of 1 nM had relatively large radioprotective effects on normal cells and tissues, but did not protect tumour cells to any significant extent. In normal clonogenic cells, where it was possible to study delayed reproductive death in the progeny, the effects of both MAO inhibitors was to prevent delayed death in progeny, with clorgyline being slightly less effective This indicates that *l*-deprenyl acts as a genome-stabilising agent. To our knowledge, this is the first report of a radioprotective agent that can prevent one of the manifestations of genomic instability.

Since exposure to *l*-deprenyl protected the normal uroepithelial explants and also resulted in significant increases in Bcl-2 protein induction, it is possible that *l*-deprenyl protects these normal human uroepithelial explants via the expression of this protein. Bcl-2 has been widely documented as an inhibitor of the apoptotic cascade (see Sentman *et al*, 1991).

Of the four irradiated cell lines studied in this work, Bcl-2 protein expression was induced by both *l*-deprenyl and, to a lesser extent, clorgyline in the normal HaCaT cell line. The more abnormal cell lines studied (HPV-G, CHO-K1 and PC3) were found to have Bcl-2 protein expression elevated to 100%. Thus, one action of *l*-deprenyl and clorgyline may be to induce this antiapoptotic pathway in the, relatively radiosensitive, normal cells. Tumour cells generally have Bcl-2 already induced, thus increasing their resistance to damage. It is interesting though to note that the antiapoptotic action of deprenyl is not the only mechanism of protection. The data in Table 1 clearly show that at 5 Gy the surviving fraction of all the cell lines whether normal or tumour is approximately 20% without *l*-deprenyl. This increases in the normal cell line, but not in the tumour lines after *l*-deprenyl treatment. Since Bcl-2 is maximally elevated in the tumour lines and induced in the normal line, the action of *l*-deprenyl cannot be solely associated with a Bcl-2-related antiapoptosis effect. Further, when the apoptosis scores at 2 and 9 days are examined, it can be seen that apoptosis is not as significantly elevated in any of the cell lines after 9 days growth in culture. It is likely that the late effect of deprenyl on the 5 Gy irradiated cells represents a different protective mechanism to that seen after 2 days. Perhaps, it is akin to the ‘neurorescue’ seen after *l*-deprenyl treatment of neuronal cells referred to in the first section. Whatever the mechanisms, these monoamine oxidase inhibitors might prove useful in protecting normal cells from therapeutic radiography in cancer patients. In this context, it is noteworthy that nontumorigenic cell lines, such as the normal HaCaT cells, normally give a relatively low plating efficiency under culture conditions, but this is significantly increased by exposure to *l*-deprenyl, presumably as a consequence of the induction of Bcl-2.

The compounds tested in this work were all MAO inhibitors and other propargylamine-derived MAO inhibitors, such as rasagiline (*N*-propargyl-[1*R*]-aminoindan) and *N*-(2-heptyl)-*N*-propargylamine (see Finberg *et al*, 1999; Huang *et al*, 1999; Maruyama *et al*, 2001) have also been shown to be neuroprotective. However, the propargylamine derivative CGP 3466 [dibenzo[*b*,*f*]oxepin-10-ylmethyl-methyl-prop-2-ynyl-amine], which is about 100 times more potent than *l*-deprenyl as a neuroprotective or neurorescuing agent is essentially devoid of inhibitory activity towards MAO (Kragten *et al*, 1998). The lack of any radioprotective effects of the MAO inhibitor pargyline would be consistent with the conclusion of others that neuroprotection is a distinct phenomenon from MAO inhibition (see eg Tatton *et al*, 1994a,1994b; Kragten *et al*, 1998). Although the propargylamine moiety might be responsible for the protection afforded by clorgyline and *l*-deprenyl, the lack of any protective effect of pargyline in our system and in some other neuronal models (see Mytilineou *et al*, 1997) would indicate it not to be a general property of all propargylamine derivatives.

The possibility that factors other than the induction of Bcl-2, which can itself prevent apoptosis in response to a number of toxic stimuli (Sentman *et al*, 1991), may contribute to the radioprotective effects of *l*-deprenyl and clorgyline requires consideration. A proportion of the cellular Bcl-2 protein is associated with the mitochondrial outer membrane (Krajewski *et al*, 1993) and the clorgyline- and *l*-deprenyl-sensitive monoamine oxidases A and B, respectively, are similarly located. Hydrogen peroxide is a product of the oxidative deamination of amines by MAO, and this may act as a potential source of toxic reactive-oxygen radicals (see, for example, Sandri *et al*, 1990). Regulation of mitochondrial and/or cytosolic reactive oxygen species (ROS) levels in mammalian cells might be mediated, in part, by the bcl-2 gene product (Hockenbery *et al*, 1993). Expression of the Bcl-2 protein prevents the induction of apoptosis by a variety of oxidative stresses, including ionising radiation, heat shock or inhibition of glutathione synthesis (Hockenbery *et al*, 1990; Zhong *et al*, 1993). It has been suggested that the bcl-2 gene product inhibits apoptosis by interacting with mitochondrial superoxide dismutase (SOD) (Itoh *et al*, 1993). Furthermore, it has been shown that various antioxidant agents (eg, *N*-acetylcysteine and glutathione peroxidase) can substitute for Bcl-2 expression in preventing factor-deprived cells from undergoing apoptosis (Hockenbery *et al*, 1993). It is, therefore, possible that the enhanced susceptibility to apoptosis that is observed in the absence of Bcl-2 expression might reflect the inability of the cell to cope with oxidative stress. Cobalt-60 gamma radiation causes the production of ROS via the radiolytic attack of water molecules and, since Bcl-2 has been implicated in inhibiting apoptosis by mechanisms that may involve SOD (Itoh *et al*, 1993), thereby rendering these species harmless, *l*-deprenyl and clorgyline could elicit their radioprotective effects via such mechanisms. Indeed, it has been reported that *l*-deprenyl increases the activity of cytoplasmic Cu/Zn-SOD, mitochondrial Mn-SOD and catalase in mouse brain (see Thiffault *et al*, 1995). However, this mechanism is implausible because of the efficacy of very low doses of deprenyl and clorgyline and the lack of an increase in effect with MAOI dose.

The most likely and very interesting explanation is that these MAOIs interfere with the induction or transmission of genomic instability in cells. Genomic instability is known to involve a persistent increase in cellular oxidative stress, and to be associated with irradiation of normal cells (reviewed in Mothersill and Seymour, 2001). Such a mechanism would explain the ability of *l*-deprenyl to reduce delayed reproductive death (lethal mutations), shown in Table 2.

To summarise, *l*-deprenyl and clorgyline show clear evidence *in vitro* of radioprotection of normal cell lines and normal tissue explants. The mechanisms are not wholly clear, but evidence is presented for one early mechanism operating via an antiapoptotic pathway involving bcl-2 induction in normal cells and tissues. A second mechanism may become important at higher doses and late time points.

These agents may be worthy of further consideration as radioprotective agents for normal tissue in the clinic.
